# Tree Reconciliation Methods for Host-Symbiont Cophylogenetic Analyses

**DOI:** 10.3390/life12030443

**Published:** 2022-03-17

**Authors:** Ran Libeskind-Hadas

**Affiliations:** Kravis Department of Integrated Sciences, Claremont McKenna College, 888 N. Columbia Avenue, Claremont, CA 91711, USA; rhadas@cmc.edu

**Keywords:** cophylogenetics, coevolution, reconciliation, hosts and symbionts

## Abstract

Phylogenetic reconciliation is a fundamental method in the study of pairs of coevolving species. This paper provides an overview of the underlying theory of reconciliation in the context of host-symbiont cophylogenetics, identifying some of the major challenges to users of these methods, such as selecting event costs and selecting representative reconciliations. Next, recent advances to address these challenges are discussed followed by a discussion of several established and recent software tools.

## 1. Introduction

In the *Origin of Species*, Darwin wrote:

I can understand how a flower and a bee might slowly become, either simultaneously or one after the other, modified and adapted in the most perfect manner to each other, by the continued preservation of individuals presenting mutual and slightly favorable deviations of structure [1].

While Darwin was prescient, the systematic study of coevolution only blossomed with the advent of DNA sequencing and computational methods, leading to a hive of activity in this field in recent years. New cophylogenetic methods, and the software tools that implement them, continue to provide clearer insights into the histories of systems of linked taxa such as the flower-bee mutualism described by Darwin, hosts and parasites, and others.

Existing cophylogenetic methods can be broadly classified as *global-fit* and *event-based* methods. Global-fit methods compute a numerical statistic that evaluates the level of congruence between two phylogenetic trees. Legendre et al. describe one such test that was implemented in the ParaFit tool [2] and Balbuena et al. described a related method implemented in their PACo tool [3]. In contrast to global-fit approaches, event-based methods provide coevolutionary scenarios called *reconciliations*. Given two phylogenetic trees and an association of their tips (extant taxa), a reconciliation is a mapping of the symbiont tree into the host tree that seeks to explain their evolutionary histories.

Each approach has its merits and limitations. The interested reader is referred to surveys by Charleston and Libeskind-Hadas [4] and Filipiak et al. [5] and an edifying discussion by Hayward et al. [6]. However, in many contexts, event-based reconciliation approaches are preferred, or even required, because they offer actual evolutionary scenarios rather than a single numerical score. Moreover, many of the limitations of event-based methods [3] have been mitigated in recent years by new techniques and algorithms. This paper, therefore, addresses the current state-of-the-art in event-based methods for host-symbiont tree reconciliation and the software tools that implement them.

## 2. The Phylogenetic Reconciliation Problem

In this discussion, the two phylogenetic trees are referred to as the *host tree*, *H*, and the *parasite tree*, *P*, recognizing that the parasite tree could, in fact, be any type of symbiont (e.g., parasite, mutualist, or commensalist) [7]. For the sake of simplicity, assume that both phylogenetic trees are fully resolved, meaning that each non-tip node has exactly two children. The discussion will return later to the more general case of non-binary trees, i.e., trees with polytomies (aka multifurcations).

Additionally, it is assumed that the two trees are undated, meaning that only the topologies of the trees are known but there is no time (aka date) information on the branches or nodes. This issue is revisited later in the paper. It is assumed that all extant taxa of the two trees are known and, thus, each tip is labeled with the name of a unique species.

Finally, an association of the tips is assumed to be known, mapping each tip of the parasite tree to one or more tips of the host tree. For the sake of clarity of exposition, assume that each parasite tip is associated with exactly one host tip and let ϕ denote the mapping of parasite tips to host tips. Note that this assumption permits two or more distinct extant parasite species to map to the same extant host species but it precludes *multi-host parasites* in which one extant parasite is found on multiple extant hosts. This issue is revisited later as well.

### 2.1. Reconciliations

A reconciliation of the parasite and host trees maps each node of the parasite tree to a node or branch of the host tree. The reconciliation must, of course, be consistent with the given tip associations. Therefore, letting L(P) and L(H) denote the set of tips (aka leaves) of the parasite and host trees, respectively, the tip associations are given by a function ϕ:L(P)→L(H). Let V(P) and V(H) denote the set of all nodes (aka vertices) of the parasite and host trees, respectively, (including both the tips and the internal nodes) and let E(P) and E(H) denote the set of branches (aka edges) of the two trees. Formally, a reconciliation is a function Φ that maps each node V(P) of the parasite tree *P* to a node or edge of the host tree, i.e., Φ:V(P)→V(H)∪E(H). This mapping function must satisfy a few basic requirements. First, Φ must be identical to the given tip mapping ϕ for the tips of *P*, i.e., for each tip *p* of the parasite tree, Φ(p)=ϕ(p). Additionally, Φ must be consistent with ancestral relationships in the two trees. Specifically, if Φ maps a non-tip parasite node *p* to a host node *h* or host edge *e*, then neither of the two children of *p* can be mapped to an edge or node that is ancestral to *h* or *e*. There are other ways of formalizing the definition of a reconciliation but the various definitions are generally functionally equivalent.

### 2.2. Events

**Figure 1:**
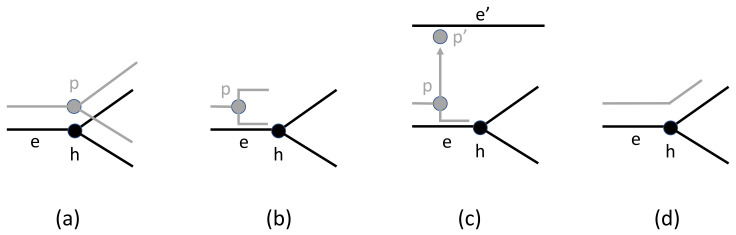
The four types of events. The fragment of the host tree is indicated in black and the fragment of the parasite tree is indicated in gray. (**a**) Cospeciation, (**b**) duplication, (**c**) host switch, and (**d**) loss.

A reconciliation induces cospeciation, duplication, host switch (aka transfer), and loss (aka extinction) events. A cospeciation event arises when a parasite node *p* speciates contemporaneously with an associated host node *h*, i.e., the reconciliation Φ maps *p* to *h* and maps the two children of *p* to nodes or edges in each of the two subtrees of *h* (Figure 1a). A duplication event arises when a parasite node speciates on an edge *e* of the host tree, i.e., non-contemporaneous speciation of a parasite and host (Figure 1b). A host switch event arises when Φ maps *p* to an edge *e* of the host tree, one of the children of *p* is mapped to *e* or one its descendants, and the other child, $p^{\prime }$, is mapped to an edge $e^{\prime }$ of the host tree that is not ancestrally related to *e* (Figure 1c). The edge *e* is said to be the *take-off site* and the edge $e^{\prime }$ is called the *landing site* of the host switch. Although the trees are generally assumed to be undated, such a host switch implies that the take-off and landing-sites are contemporaneous. Such implicit time relationships are discussed in the next section. Finally, loss (extinction) events arise as follows: Consider a parasite node *p* mapped to a host node *h* (or host edge *e* where *h* is the starting node of *e*; the endpoint of *e* closest to the root) and a child $p^{\prime }$ of *p* mapped to host node $h^{\prime }$ (or host edge $e^{\prime }$ where $h^{\prime }$ is the ending node of $e^{\prime }$; the endpoint of $e^{\prime }$ furthest from the root). The path from *h* to $h^{\prime }$ in *H* may pass through zero or more nodes of *H*. Each such node corresponds to a loss event in this reconciliation (Figure 1d).

### 2.3. Maximum Parsimony and Optimal Reconciliations

In general, reconciliation is performed under a maximum parsimony framework (Reconciliation using a maximum likelihood objective function is also theoretically possible, but computationally intractable). In this approach, each type of event has an associated cost and a reconciliation of minimum total cost is sought. More precisely, if the event costs for cospeciation, duplication, host switch, and loss are denoted $C_{c},C_{d},C_{s}$ and $C_{\ell }$ and a reconciliation Φ induces *c*, *d*, *s*, and *ℓ* cospeciation, duplication, host switch, and loss events, respectively, then the cost of that reconciliation is $\mathrm{cost}\left(\Phi \right)=C_{c}\times c+C_{d}\times d+C_{s}\times s+C_{\ell }\times \ell$. For a given set of costs, a reconciliation Φ is sought that minimizes this quantity. Such a reconciliation is called an *optimal reconciliation* or, more commonly, a *maximum parsimony reconciliation (MPR)* to indicate that it is optimal with respect to the maximum parsimony criterion. The vector (c,d,s,ℓ) denoting the number of events of each type is called the *event count vector* for that reconciliation.

**Figure 2:**
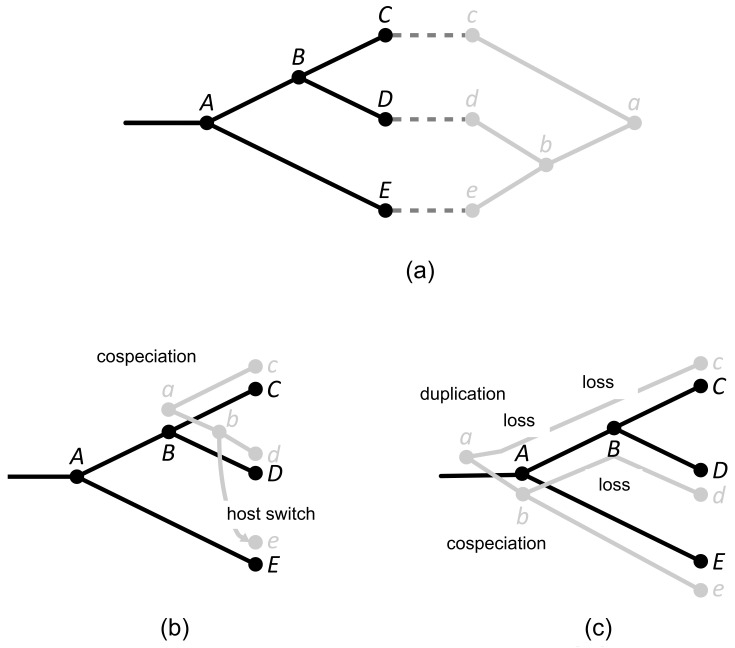
(**a**) A host tree (black), a parasite tree (gray), and a tip association indicated by dashed lines between tips. (**b**,**c**) Two different reconciliations annotated with their induced events. The reconciliation in (**b**) is optimal when cospeciation has cost 0 and all other events have cost 1. The reconciliation in (**c**) is optimal when cospeciation has cost 0, duplication and loss have cost 1, and host switch has cost 5.

For example, in Figure 2, if cospeciation has cost 0 and duplication, host switch, and loss all have cost 1 then the reconciliation in (b) has cost 1 (one host switch), whereas the reconciliation in (c) has cost 4 (one duplication, 3 losses). It is not difficult to verify that the reconciliation in (b) is optimal in this case. On the other hand, if cospeciation has cost 0 and duplication, host switch, and loss have costs 1, 4, and 1, respectively, then both reconciliations have cost 4 and these can be verified to be optimal with respect to these event costs. Finally, if cospeciation has cost 0, duplication and loss have cost 1, and host switch has cost 5, then the reconciliation in (b) has cost 5 wheres the reconciliation in (c) has cost 4 and is optimal for those event costs. This small example demonstrates that (1) optimality depends on the event costs and (2) there can be multiple optimal solutions for a single set of event costs.

It is important to note that event costs have no units; event costs are only meaningful in terms of their relative magnitudes. Therefore, choosing event costs of 0,1,2,1 for cospeciation, duplication, host switch, and loss will yield exactly the same results as choosing costs of 0,100,200,100 for those events.

It should also be noted that concept of a reconciliation is predicated on mapping one tree (e.g., the parasite tree) into the other (e.g., the host tree). In the context of host-symbiont relations, this notion is natural since it is assumed that the symbiont evolves in response to the host rather than vice versa. However, in some cases it may be less clear which tree should be mapped into the other. In general, the reconciliation problem is asymmetric in the sense that the events are induced by the mapping of *P* into *H*. If, instead, *H* is mapped into *P*, different events will be induced and, in general, a most parsimonious mapping of *P* into *H* has no relationship to a most parsimonious mapping of *H* into *P*.

Finally, the reconciliation problem also arises in study of the evolution of homologous genes evolving within species as discussed in Section 5.

## 3. The Challenges of Reconciliation

While phylogenetic reconciliation is a powerful concept, it gives rise to several challenges. This section outlines some of the most salient challenges and methods that have been recently developed to address them.

### 3.1. Event Costs

As noted above, different event costs may give rise to different MPRs (optimal reconciliations), potentially resulting in different conclusions about the evolutionary histories of the pair of taxa. Choosing appropriate event costs can be challenging. Indeed, this is one of the principle objections to event-based approaches [3,8]. In many studies, conclusions are made based on a single set of event costs or a small sample of different event costs. Often, these event costs are simply the default values in the particular software package used to perform the analysis. Commonly, cospeciation is set to have cost 0 and duplication, host switch, and loss events are assigned positive costs. In this way, if the host and parasite trees are identical and each tip of the parasite tree is associated with the corresponding tip of the host tree, the MPRs will have cost 0. All other reconciliations will have positive cost. In some analyses, cospeciation is set to have cost −1 and all other events have costs 0. In this way, a maximum parsimony reconciliation is one that has the largest number of cospeciation events. In some studies, researchers have simply sampled several different combinations of event costs based on their estimates of the prevalence of those events. For example, commonly cospeciation is set to cost 0, loss is set to cost 1, and then duplication and host switch costs are sampled relative to those costs. In any case, the choice of event costs can be a significant challenge in practice.

**Figure 3:**
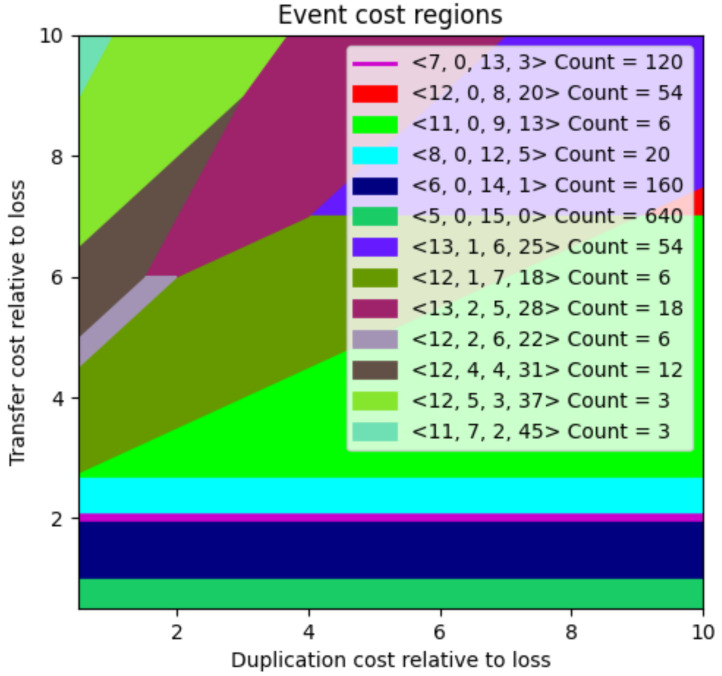
The event cost landscape for the *Vidua* dataset is comprised of 33 host and 21 parasite species. The cost of a loss event is fixed to 1 and the *x*- and *y*-axes show duplication and host host switch costs, respectively, ranging from 0.5 to 10. Each color-coded region represents a set of event costs that give rise to the same set of MPRs. Thus, it suffices to choose just one set of event costs in a region. For each region, the legend shows a vector of the form 〈c,d,t,ℓ〉 which represents the number of cospeciation, duplication, host host switch, and loss events for solutions in that region. The “Count” indicates the number of distinct MPRs for any set of event costs in that region.

To address this challenge, Libeskind-Hadas et al. developed a set of algorithms called *xscape*. One of those algorithms, *costscape*, provides an overview of the solution space over a range of possible event costs [8]. In this approach, cospeciation events are assumed to be “null events” and have cost 0 while duplications, host switches, and losses are assumed to have strictly positive costs. For a given pair of trees and their tip associations, the costscape algorithm efficiently computes and displays a summary of the solution space with respect to loss events normalized to cost 1 and duplication and host switch events varying over a user-specified range as shown in Figure 3 for the *Vidua* dataset from [9] (Recall that event costs have no units, so only the relative magnitude of the event costs is important. There is, therefore, no loss of generality in setting loss events to have cost 1). In this plot, each color-coded region corresponds to a set of event costs that give rise to exactly the same set of MPRs. Formally, these regions are called *equivalence classes* because the solutions in each region are equivalent. The legend provides additional information on each region. For example, for the light blue region in the figure, the legend associates the event count vector (8,0,12,5) and a count of 20 meaning that for event costs selected from the blue region there are exactly 20 distinct MPRs and these reconciliations have several cospeciation, duplication, host switch, and loss events equal to 8,0,12 and 5, respectively.

Because all choices of event costs in the same equivalence class give rise to the same solutions, the costscape permits the researcher to systematically select event costs over a range of plausible event costs rather than haphazardly sampling event costs. As discussed in Section 4, the costscape algorithm is implemented in the eMPRess software package.

### 3.2. Dating and Time-Consistency

Recall that the host and parasite trees are assumed to be undated because accurate complete dating information is, in general, difficult or impossible to obtain. As discussed in Section 4, some software packages allow the user to add annotations with the relative times (aka dates) of nodes in one or both trees. This section addresses the case of undated trees since it is the most general.

**Figure 4:**
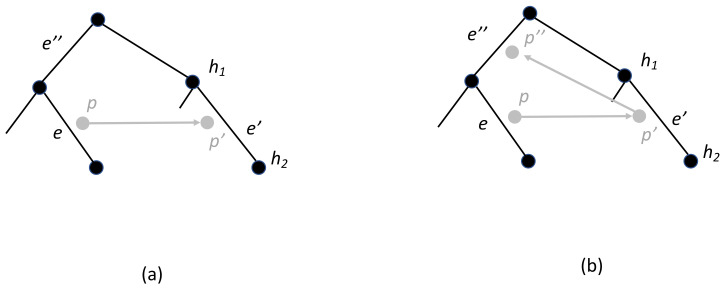
(**a**) Node *p* must occur before node $h_{2}$. (**b**) Node *p* must occur before node $p^{\prime \prime }$ but node $p^{\prime \prime }$ must occur before node *p*, a contradiction.

Phylogenetic trees induce implicit constraints on the order of tree nodes. Specifically, a node *v* in a tree must occur earlier in time than any of its descendants. Reconciliations impose additional implicit constraints on the order of nodes as shown in Figure 4a. In this example, parasite node *p* mapped to host edge *e* must occur before host node $h_{2}$ because *p* has a child, $p^{\prime }$, mapped to the edge $e^{\prime }$ between $h_{1}$ and $h_{2}$ via a host switch. Such time relationships are implied by a reconciliation rather than by the trees themselves.

Some sets of events in a reconciliation may be infeasible because they induce incompatible time constraints. In particular, a sequence of host switch events can give rise to these kinds of incompatibilities. Continuing with the previous example, if $p^{\prime }$ has a child $p^{\prime \prime }$ such that $\Phi \left(p^{\prime \prime }\right)=e^{\prime \prime }$ and $e^{\prime \prime }$ is ancestral to *e*, then a contradiction arises as shown in Figure 4b. At issue here is that the host switch involving *p* and $p^{\prime }$ is valid because $e^{\prime }$ is not ancestrally related to *e* and, similarly, the host switch involving $p^{\prime }$ and $p^{\prime \prime }$ is valid because $e^{\prime \prime }$ is not ancestrally related to $e^{\prime }$. However, while these switches are individually valid, this sequence of host switch events causes an inconsistency. In general, a reconciliation in which parasite node $p_{1}$ is ancestral to parasite node $p_{2}$ implies that $\Phi (p_{2})$ is not ancestral to $\Phi (p_{1})$ is said to be *time-consistent*. This will be referred to, however, as *weak time-consistent* to contrast it with an even stronger version of time-consistency, described next.

**Figure 5:**
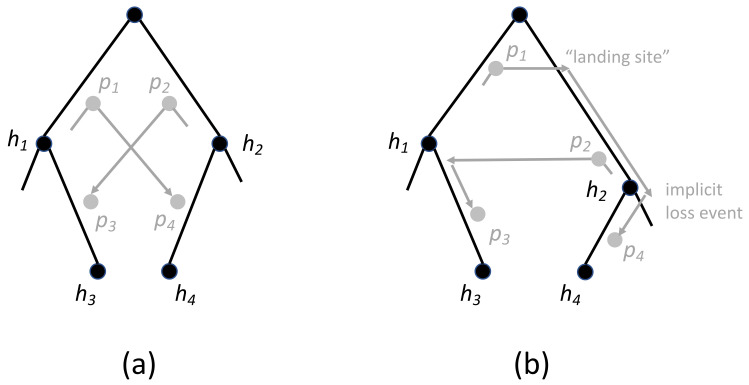
(**a**) A fragment of a reconciliation that is infeasible due to incompatible constraints. In this mapping, node $p_{1}$ occurs before $h_{1}$ and node $p_{2}$ occurs before $h_{2}$. In addition, $h_{2}$ must occur before $p_{1}$ in order for edge $\left(h_{2},h_{4}\right)$ to be contemporaneous with $p_{1}$. It follows that $h_{2}$ must occur before $h_{1}$. Analogously, $h_{1}$ must occur before $h_{2}$, resulting in a contradiction. (**b**) A “corrected” version of the reconciliation requires the addition of at least one implicit loss event that was not accounted for in the original reconciliation.

A more complicated timing issue arises in the example shown in Figure 5a. In this case, there is no violation of weak time-consistency, but the fragment of the reconciliation shown here is not possible since it induces two mutually incompatible timing constraints. The reconciliation can be “corrected” by introducing additional loss events as shown in Figure 5b, but those loss events were not posited, and thus not accounted for, in the original reconciliation. By adding those implicit losses in after-the-fact, the cost of the reconciliation increases and it is no longer necessarily a minimum cost reconciliation. Thus, while this reconciliation is weak time-consistent, it is not *strong time-consistent*. Specifically, a reconciliation is strong time-consistent if it induces no timing incompatibilities.

The problem of finding weak or strong time-consistent reconciliations in undated trees has been shown to be computationally intractable, i.e., NP-hard [10,11]. Therefore, many tools use efficient polynomial-time dynamic programming algorithms [12,13] to compute MPRs with the caveat that these reconciliations are not guaranteed to be weak or stong time-consistent. However, a reconciliation can be tested for weak or strong time-consistency and some tools, such as eMPRess (described in Section 4), report the type of time-consistency in the MPR.

It should be noted that in the case that the host tree is not fully sampled, it is theoretically possible for a parasite lineage to host switch to an unsampled species outside of the constructed host tree. Subsequently, that parasite lineage may host switch back to the sampled host tree. This type of host switch in-and-out of the host tree has been called *host switch from the dead* [14]. In this setting, there is no distinction between weak and strong time-consistency because a parasite node can effectively be host switched forward in time by host switching out of the host tree to an unsampled lineage and then, later, host switch back to the host tree. However, in many cophylogenetic studies, the host species is assumed to be completely sampled and the effect of extinct species is not considered.

### 3.3. Selecting from the Large Space of MPRs

Another challenge that arises in tree reconciliation is the potentially very large number of MPRs. It is known that the number of MPRs can grow exponentially in the size of the two trees [15] and that these reconciliations can differ substantially [16]. In some datasets with at most 100 tips in each tree, the number of MPRs for a given set of event costs can exceed $10^{100}$ [17] which is comparable to the number of particles in the known universe!

Several different approaches and algorithms have been developed to address this issue. Wang et al. proposed three different ways of grouping reconciliations; that is, three different ways of defining equivalence classes over the space of reconciliations [18]. The first approach is to partition the set of MPRs with respect to their event count vectors as in in the costscape approach described previously, i.e., two reconciliations are considered *event-vector equivalent* if they have the same event count vectors. The second approach is to define two MPRs to be *event-partition equivalent* if for each parasite node, the type of event associated with *p* (cospeciation, duplication, or host switch) is the same in both reconciliations. Note that two different reconciliations can be event-partition equivalent. For example, a parasite node *p* may be mapped to host node $h_{1}$ as a cospeciation event in one reconciliation and mapped to a different host node $h_{2}$ as a cospeciation event in the other reconciliation. In other words, this notion of equivalence only considers the *types* of events associated with each parasite node but not the specific *location* to which it is mapped. Finally, two reconciliations are said to be *cospeciation-duplication equivalent* if they are event-partition equivalent and, additionally, they agree exactly on the locations of all cospeciation and duplication events (but not necessarily on host switch events), i.e., if two reconciliations are cospeciation-duplication equivalent then for each parasite node *p* mapped to a host node *h* as a cospeciation event in one reconciliation, that node is also mapped to the same node *h* as a cospeciation in the other reconciliation *and* if *p* is mapped to a host edge *e* as a duplication in one reconciliation then it is mapped to the same edge *e* as a duplication in the other reconciliation.

Wang et al. showed that these three equivalence classes can be computed by efficient algorithms and implement those algorithms in their Capybara tool [18], described in more detail in Section 4. In particular, their algorithms compute the number of MPRs in each equivalence class and enumerate them. While these approaches seek to partition the large space of solutions into smaller groups, the number of reconciliations in these equivalence classes can still be extremely large.

In an effort to provide a single “best representative” reconciliation from the potentially very large space of MPRs, Nguyen et al. [19] defined the notion of a *median reconciliation*. Recall that a median of any set is an element that minimizes the sum of the distances to all other elements in that set. For example, in the set of numbers {1,2,5,6,10} (where the distance between two elements is understood to be the absolute value of their difference), the median element is 5. In the set {1,2,5,6,9,10} both 5 and 6 are median elements. In general, medians are not unique.

In the context of reconciliations, Nguyen et al. proposed the use of the *symmetric set distance* metric [19]. Given two reconciliations $R_{1}$ and $R_{2}$, the distance between those reconciliations is defined to be the number of events that are found in one reconciliation but not the other, i.e., the number of events in $R_{1}$ but not in $R_{2}$ plus the number of events that are in $R_{2}$ but not in $R_{1}$. In this context, an event is characterized by a parasite node *p*, the host node *h* or edge *e* to which *p* is mapped in that reconciliation, the type of the event (cospeciation, duplication, or host switch) and the mapping of the children of *p*. Loss events can also be considered in this distance metric. For example, consider the case that $R_{1}$ maps parasite node *p* to host edge *e* as a host switch event and the left and right children of *p*, $p_{1}$ and $p_{2}$, are mapped to host edges $e_{1}$ and $e_{2}$. Additionally, in $R_{2}$, *p* is also mapped to *e* but as a duplication event. These two events are different and thus each one contributes one to the distance. If, instead, in $R_{2}$ node *p* is mapped to *e* as a host switch, $p_{1}$ is mapped to $e_{1}$ but $p_{2}$ is mapped to a host edge $e_{3}\neq e_{2}$, these events are also considered to be different; again each one contributes one to the distance. In other words, this definition of distance is very “fine-grained” in the sense that any difference in events contributes to the distance between the two reconciliations.

Nguyen et al. showed that even though the number of MPRs can be exponential in the number of nodes in the two trees, a median reconciliation with respect to the aforementioned symmetric distance metric can be computed by an efficient algorithm [19]. The key to this surprising result is the use of a compact representation of the large space of reconciliations called the *reconciliation graph* [20]. Many subsequently developed efficient algorithms related to reconciliations are also based on this this graph representation.

A median reconciliation is, intuitively, an appealing choice of a single representative for a potentially large space of MPRs. However, in some cases when the space is large and diverse, the number of medians can itself be extremely large [21]. In that case, it is unlikely that any one single reconciliation can adequately represent the entire space.

**Figure 6:**
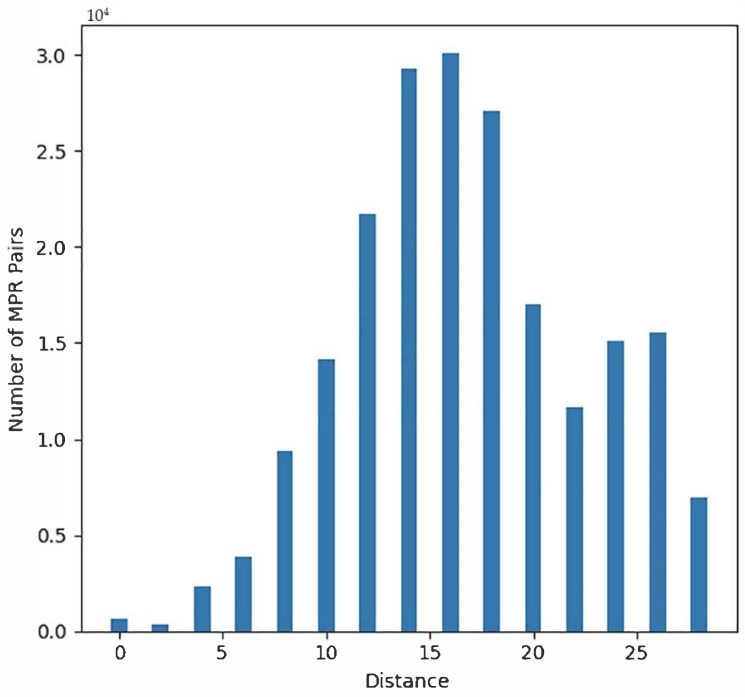
A pairwise distance histogram for the *Vidua* dataset using event costs of 0.9 and 2.0 for duplication and host switch, respectively, relative to cost of 1.0 for loss. The upper-left corner of the plot shows that the *y*-axis scale is $10^{4}$ in this case. The *x*-axis indicates the distance between pairs of MPRs. In this case, there are 640 MPRs total, and thus 204,480 distinct pairs of MPRs. Every pair of MPRs is counted exactly once in this plot. In addition to the distinct pairs of MPRs, for calibration all 640 MPRs paired with themselves are included, which accounts for the 640 pairs of MPRs at distance 0 as indicated at x=0. The histogram indicates that there are over $1.5\times 10^{4}$ pairs of MPRs that differ in exactly 20 events and over $0.5\times 10^{4}$ MPRs that differ in as many as 28 events.

Santichaivekin et al. described an efficient algorithm for visualizing the diversity of MPRs [17]. For a given set of MPRs, the *pairwise distance histogram* is a histogram of the distances between all pairs of reconciliations in that set with respect to the symmetric set distance described above (where losses are included in the distance in addition to cospeciation, duplication, and host switch events). Santichaivekin et al. showed that despite the fact that the number of MPRs can grow exponentially with the number of nodes in the two trees, the pairwise distance histogram can be computed exactly by an efficient algorithm using the reconciliation graph representation mentioned above. Figure 6 shows an example of a pairwise distance algorithm for the *Vidua* dataset [9]. Pairwise distance histograms are implemented in the eMPRess tool described in Section 4.

The pairwise distance histogram provides an overview of the distribution of the space of MPRs. In the event that the distribution is narrow, choosing a single representative such as the median may suffice. However, for wide or multi-modal distributions, it may be more appropriate to select several representative MPRs. Ozdemir et al. [16] and Mawhorter and Libeskind-Hadas [22] gave algorithms for clustering the space of MPRs where the reconciliations in each cluster are relatively similar with respect to the symmetric set distance and reconciliations in different clusters are relatively dissimilar. These algorithms differ in their clustering criteria, but share the feature that the user can choose the desired number of clusters and the algorithms will produce one median reconciliation from each cluster.

As in most applications involving clustering, there is generally no rule for determining the “correct” number of clusters. However, if the distribution of distances is multimodal, as in the case of the histogram in Figure 6, it is likely that there are at least two natural clusters: MPRs within the same cluster will typically have relatively lower pairwise distances (corresponding to the first mode) than will pairs between different clusters (corresponding to the second or higher modes). In practice, one may wish to experiment with different numbers of clusters. When adding an additional number of clusters does not significantly change the distribution of pairwise distances, one may conclude that the natural clusters have been found and subdividing the space into additional clusters is no longer useful. The hierarchical clustering algorithm developed by Mawhorter and Libeskind-Hadas is implemented in the eMPRess tool described in Section 4.

### 3.4. The Strength of Evidence for Tree Congruence

While maximum parsimony reconstructions attempt to infer evolutionary events, the reconstructions and their numerical scores alone do not suffice to address the question of whether or not the level of congruence indicated by the numerical reconciliation score is due to chance. Congruence is usually determined by performing random permutation tests. Specifically, let *c* denote the maximum parsimony cost for the given pair of trees and tip mapping. The parasite tree *P* or the tip mapping ϕ is randomized some number of times, denoted *n*, and the maximum parsimony cost is recomputed for each randomized instance. The fraction of random instances whose cost is less than or equal to *c* is counted and denoted *r*. Then, the quantity $\frac{r\;+\;1}{n\;+\;1}$ serves as an empirical *p*-value. In other words, the null hypothesis that the two trees and their tip association are concordant due to chance can be rejected if the *p*-value is sufficiently small (e.g, 0.05 or 0.01). As described in the next section, several reconciliation tools compute empirical *p*-values using this type of randomization process.

## 4. Algorithms and Tools

Many software tools were developed for the tree reconciliation problem in the context of hosts and symbionts. Rather than enumerating all of them and comparing their features, this section describes the three most established and widely used packages; TreeMap [23], CoRE-PA [24], and Jane [25]); as well as two newcomers with important and novel features, Capybara [18] and eMPRess [26] (Jane and eMPRess were developed by the author’s research group). Henceforth, these are denoted as the “five software packages under consideration.” All of these software packages have graphical user-interfaces and some also offer alternative command-line interfaces which can be useful when embedding reconciliation in an analysis pipeline or in developing a script to automate the analysis of a large number of data sets. As discussed in the next section, reconciliation can also be performed using gene tree-species tree software tools such as Ranger-DTL [27], Mowgli [28], Eucalypt [29], ecceTERA [30], but may of these tools lack graphical user-interfaces or other features that are useful in cophylogenetic studies.

The five software packages under consideration differ in the way that they handle event costs. TreeMap identifies reconciliations that are *Pareto-optimal*, analogous to finding a representative reconciliation in each of the equivalence classes of costscape described in Section 3.1. TreeMap uses a heuristic to identify these representative reconciliations but does not generate all of them using an efficient algorithm. In contrast, Capybara and eMPRess use efficient exact algorithms to identify these equivalence classes. Capybara will automatically produce a list of reconciliations from each such event count equivalence class while eMPRess displays the equivalence classes graphically and allows the user to obtain a single median reconciliation from each class or cluster the equivalence class and obtain a median reconciliation from each cluster. While most of these packages allow the user to select all four event costs, eMPRess fixes the cospeciation cost to zero under the assumption that cospeciation is a “null” event in a reconciliation. CoRE-PA automatically selects event costs by optimizing a mathematical objective function but it is not clear if those event costs are biologically realistic. Jane requires that the user select their own event costs which, as noted earlier, can be challenging. To help mitigate this issue, Jane allows the user to select ranges of event costs when computing empirical *p*-values.

These software packages assume that trees are undated but two of the packages, CoRe-PA and Jane, offer support for dating information in the form of “time zones.” The user may partition the nodes of a tree into an arbitrary number of groups numbered 1 through *k*. All nodes in group *i* occur before nodes in group i+1, for 1≤i<k. Placing each node in a distinct time zone effectively becomes a complete dating of the tree and the timings induced by the reconciliations will satisfy these time zone constraints. This is particularly relevant for host switch events: If a host switch occurs from an edge within a given time zone then this take-off site and the landing site are constrained to be in the same time zone.

Unresolved trees (non-binary trees) pose an additional challenge, since there are many ways to resolve a given non-binary node (aka polytomy or multifurcation). CoRE-PA and Jane allow for non-binary trees and use heuristics to find resolutions that result in good, but not necessarily optimal, reconciliations. Jane, for example, samples a subset of all possible resolutions, finds a good reconciliation for each one, and reports the resolutions and reconciliations that are best among all samples.

The maximum parsimony framework is a widely used mathematical proxy for biological phenomena but restricting our attention to mathematically optimal mappings may be too restrictive. Capybara allows the user to enumerate a user-defined number, *k*, of reconciliations. It then enumerates the *k* least-cost reconciliations, breaking ties arbitrarily, and thus allowing for sub-optimal reconciliations to be enumerated. It should be noted that in many cases, the number of optimal reconciliations (MPRs) alone is extremely large and thus even enumerating them is impractical.

TreeMap, CoRE-PA, and Jane all support randomization tests to compute significance tests as described in Section 3.4.

These software packages differ in their running times. TreeMap and Jane guarantee strong time-consistent solutions but, since this problem is computationally intractable (NP-hard), these tools use heuristics that result in solutions that are not necessarily optimal. In TreeMap, the user can control running time by setting a limit on the number of host switch events. In contrast, Jane uses a metaheuristic called a *genetic algorithm* that samples a subset of the possible time-consistent solutions and finds those with the best cost which, again, may not be optimal. CoRE-PA, Capybara, and eMPRess all use similar very fast reconciliation algorithms that guarantee optimal cost but are not guaranteed to be time-consistent. The eMPRess tool uses an efficient algorithm to analyze the reconciliations that it finds and indicates whether they are weak time-consistent, strong time-consistent, or neither.

The relatively new Capybara and eMPRess tools have unique features that seek to address the significant challenge of finding representative reconciliations in the potentially very large space of all MPRs. As noted in Section 3.3, their approaches are quite different: Capybara defines three different notions of equivalence of MPRs and generates a user-specified number of MPRs in each equivalence class. The reconciliation and per-MPR generation step are fast, but there can be a very large number of MPRs in each equivalence class. In contrast, eMPRess offers the choice of selecting a single median MPR or clustering the space of MPRs and selecting a median to represent each cluster. This clustering algorithm can be slow for large data sets.

Finally, Jane is unique in its support for multi-host parasites, i.e., extant parasites that are found on multiple extant host species. A fifth event, called *failure to diverge* [31] is used to generate reconciliations in such cases. Jane also has support for *preferential host switching* [31], allowing the cost of the host switch event to be dependent on the distance from the take-off site to the landing site in the host tree as well as support for limiting the maximum distance allowed for a host switch event.

In summary, all of the software packages described here provide powerful tools for evaluating host-symbiont relationships. Some tools have unique features that may be of use in particular cases. For example, if time-consistent solutions are imperative, TreeMap and Jane may be the right choice, although eMPRess will also report whether or not each MPR that it finds is time-consistent. On the other hand, the time-consistent restriction results in slower algorithms which, in turn, cause TreeMap and Jane to return potentially suboptimal (larger than minimum cost) solutions whereas the solutions found by the other tools are guaranteed to have minimum cost. When event costs are difficult to estimate, TreeMap and eMPRess offer “Pareto-optimal” solutions—solutions that are optimal over a range of different event costs. When the large space of solutions is difficult to assess, Capybara and eMPRess offer unique tools for finding representative reconciliations in those large spaces.

## 5. Reconciliation of Gene Trees and Species Trees

As noted in the previous section, the reconciliation problem also arises in the context of reconstructing the histories of gene families and species. In this context, the objective is to map a gene tree into a species tree. In the DTL model, the modeled events are speciation, gene duplication, horizontal gene transfer, and gene loss which are mathematically analogous to cospeciation, duplication, host switch, and loss, respectively, in the context of symbionts and their hosts. Therefore, the maximum parsimony reconciliation problem for genes and species in the DTL is identical to the reconciliation problem discussed here for symbionts and hosts. Many of the techniques and tools described in this paper benefited from advances in reconciling gene trees and species trees [11,12,27,32,33]. In the context of genes and species, other events may also be modeled which typically do not arise in cophylogenetic studies, such as population effects due to incomplete lineage sorting (ILS) [34].

A number of other advances were made in the community of researchers studying the inference of gene trees with species trees. For example, since maximum parsimony reconciliation can result in many equally optimal solutions, contributions have been made on probabilistic models that allow for the integration over these large solution spaces. In some cases, gene trees may be erroneous due to inadequate signal strength in the sequence data, requiring gene tree correction. Szöllősi et al. surveyed of some of the issues and results [35] that are particularly relevant in the context of inferring the relationships between gene trees and species trees.

## 6. Conclusions and Future Work

The last several decades saw substantial advances in computational methods and tools for host-symbiont cophylogenetic analysis. This paper described some of the main advances in event-based reconciliation methods and the features and strengths of both well-established and new software tools. Several research groups continue to develop new techniques and algorithms which periodically manifest themselves in new and improved tools.

There are ample opportunities for future work and improvements to existing tools. While Capybara and eMPRess have powerful features for assessing the large and diverse space of maximum parsimony reconciliations, this problem is far from being “solved” and it is likely that there will be continuing advances here.

First, systematic studies are warranted for testing the accuracy of various phylogenetic methods. For example, simulation tools such as the one proposed by Dismukes and Heath [36] may be useful in performing these assessments using synthetically generated datasets.

Often, there are multiple candidate phylogenies for both the host and symbiont and it may be desirable to automatically select those phylogenies that have the best reconciliations. In some cases, as in systems involving hyperparasites, it is desirable to simultaneously reconcile three or more trees (e.g., host, parasite, and parasitoid). These and other challenges will undoubtedly continue to spur the development of future research and improved software tools.

The eMPRess tool (sites.google.com/g.hmc.edu/empress/home, accessed 10 March 2022) is updated periodically to address user needs and to add new features. Among the features anticipated in future releases are support for unresolved (i.e., non-binary) phylogenetic trees, time zones (for annotating the relative times of events in the two phylogenetic trees), and multi-host parasites by considering additional events such as failure to diverge [31] and incomplete host switching [37].

## Data Availability

Not applicable.
